# Reversible cerebral vasoconstriction with thunderclap headache: A case report

**DOI:** 10.1097/MD.0000000000018254

**Published:** 2019-12-10

**Authors:** Jae Young Ji, Ho Soon Jung, Sie Hyeon Yoo, Hee Dong Son, A. Joo Kim

**Affiliations:** Department of Anesthesiology and Pain Medicine, Soonchunhyang University Cheonan Hospital, Cheonan, Chungcheongnam-do, Republic of Korea.

**Keywords:** headache, reversible cerebral vasoconstriction syndrome, trigger point injection

## Abstract

**Rationale:**

Reversible cerebral vasoconstriction syndrome (RCVS) is often accompanied by thunderclap headaches. Although symptoms usually resolve spontaneously within 2 months, it can cause fatal complications, such as cerebral hemorrhage, and is difficult to differentiate from a migraine and other headaches on the basis of symptoms and Imaging study. In this case report, we explore clinical findings and appropriate treatment methods for RCVS through the case study of a female patient who experienced severe headache upon defecation

**Patient concerns:**

A 42-year-old female patient complained of a severe throbbing headache with a Numeric Rating Scale (NRS) score of 10 after defecation. The pain subsided temporarily after treatment with diclofenac 75 mg and Tridol 50 mg propacetamol 1 g, but the headache returned upon defecation; soon after, the patient complained again of regular headaches at 4 to 6-hour intervals irrespective of defecation.

**Diagnosis:**

Brain computed tomography (CT) and head and neck magnetic resonance angiography, performed during a headache episode, revealed no specific neurological findings. Blood analysis was also normal. Head and neck CT angiography, performed one month after the start of the headaches, revealed RCVS.

**Interventions:**

Treatment commenced with pregabalin (150 mg), oxycodone HCl/naloxone (10/5 mg), Alpram (0.5 mg), milnacipran (25 mg), and frovatriptan 25 mg, but there was no improvement in the headaches. The patient received bilateral trigger point injections (TPI) in the temporal muscles on four occasions at the pain clinic.

**Outcomes:**

Medication showed no effect, but after the patient received four sessions of bilateral TPI in the temporal muscles her NRS score eventually decreased from 10 to 2. The patient is currently continuing medication while still experiencing headaches at reduced intensities.

**Lessons:**

RCVS is difficult to diagnose; moreover, it is difficult differentiate RCVS from other headaches. However, as it can cause fatal complications, it should not be overlooked. It is essential to consider diagnostic treatment for all types of headaches because RCVS can be accompanied by headaches originating from other causes.

## 1 Introduction

Thunderclap headache occurs suddenly, reaching a Numeric Rating Scale (NRS) score of 7 or higher within 1 minute, and can persist from several minutes to several days.^[1]^ As thunderclap headache can lead to fatal complications, such as cerebral hemorrhage, evaluation is crucial if symptoms occur. The causes of secondary headaches are diverse; one being reversible cerebral vasoconstriction syndrome (RCVS), which is not seen commonly in clinical practice and for which symptoms usually improve within 2 months.^[2]^ However, given that radiological and hematological test results immediately after the onset of symptoms are often normal, early diagnosis can be difficult. Because of the associated risk of fatal complications, including cerebral hemorrhage, cerebral infarction, and cerebrovascular dissection—typically involving the carotid or vertebral arteries,^[3,4]^ it is important not to overlook RCVS. In this case report, we explore clinical findings and appropriate treatment methods for RCVS through the case study of a female patient who experienced severe headache upon defecation.

## 2 Case presentation

**Figure 1:**
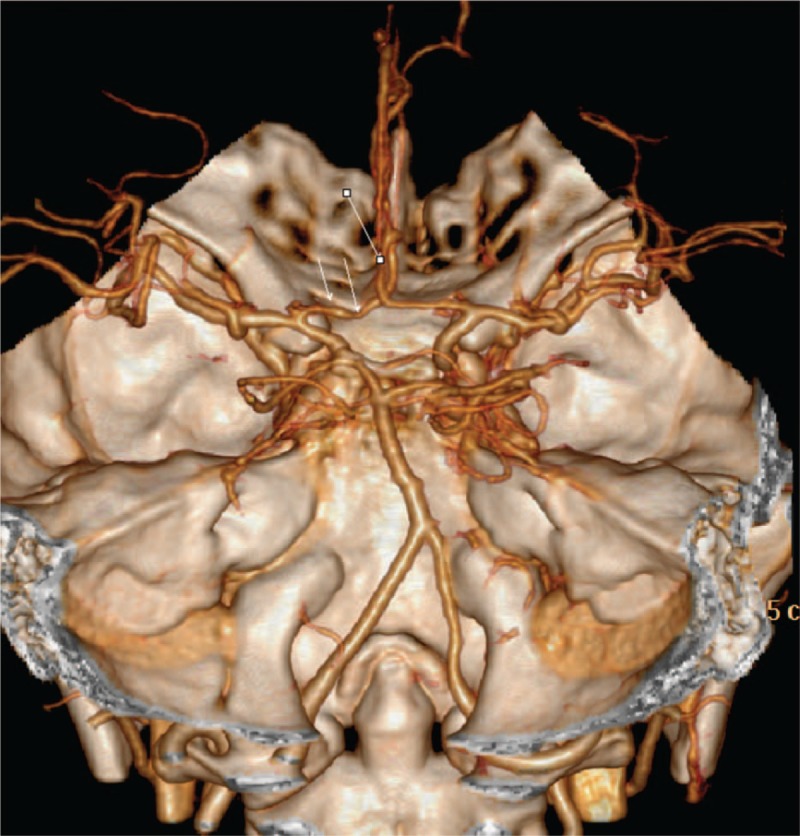
Head and Neck Computed Tomography Angiography. It showed bilateral, multifocal, reversible vasoconstriction of the anterior cerebral artery, leading to a diagnosis of RCVS.

This case report was approved by the Institutional Review Board of Soonchunhyang University Hospital (IRB No.2019-02-019), and the patient gave written informed consent for publication of this case report and accompanying images. A 49-year-old female patient with no previous history of headache visited our pain clinic complaining of headache. This patient was being treated for hyperthyroidism, major depressive disorder, and fibromyalgia. Four days after receiving laparoscopic supracervical hysterectomy for a myoma diagnosis, the patient experienced a severe throbbing, pulsating headache, with an NRS score of 10, upon defecation. At the time of the headache, the patient's blood pressure was 180/84 and heart rate was 84. The headache presented with pain throughout the whole head and was especially severe in the anterior and lateral areas. It became more severe with movement and was accompanied once by vomiting. Following the onset of the headache, the patient was administered diclofenac (Kinpoin, 75 mg) and propacetamol (Denogan, 1 g) injections, after which her pain was reduced to an NRS score of 2. However, the next day, upon defecation, the patient again complained of a headache with a visual analog scale (VAS) score of 10. Initially, the pain was only experienced upon defecation, but after 1 week, the patient started experiencing regular pain, irrespective of defecation, at 4 to 6-hour intervals. On brain CTA scans taken 30 minutes after the onset of the initial headache, there were no specific findings, and there were also no notable findings in head and neck magnetic resonance angiogram (MRA) taken the day after the headache. As the patient had experienced pain in the bilateral trapezius areas accompanied by headache, complement, antinuclear antibody (ANA), and rheumatoid factor were checked to test for polymyalgia rheumatica; however, all these tests were negative. Spinal tapping was performed to check for meningitis, but the cerebrospinal fluid (CSF) also showed no specific findings. As the patient had been previously diagnosed with depression based on psychiatric symptoms such as anxiety, insomnia, and reduced appetite as well as psychiatric examination, the findings were consistent with the possibility of somatic symptom disorder. In order to alleviate the headaches, treatment was commenced with pregabalin (150 mg), oxycodone HCl/naloxone (10/5 mg), alpram (0.5 mg), milnacipran (25 mg), and frovatriptan, but there were no signs of improvement. Three days after the onset of headaches, bilateral occipital nerve block was performed using 0.5% mepivacaine (1 ml), but headache occurred again on defecation. Nine days after headache onset, the patient complained of very severe pain on both sides of the head, and so trigger point injection (TPI) was performed in the bilateral temporalis muscles using 0.5% mepivacaine (1 ml), after which the patient showed signs of symptom relief, with pain decreasing from an NRS score of 10 to 6, and then to a score of 2 after 4 to 6 hours. The headache then recurred, and TPI was administered again in the same location, 3 days later. After the second round of TPI, the headache symptoms still persisted, but the pain intensity decreased from an NRS score of 5 to 2, and subsequently stabilized at 3–4. TPI was administered to the bilateral temporalis muscles twice more at 10-day intervals, after which the same headache pattern was maintained with an NRS score of 2. Head and neck CTA was performed again one month after headache onset (Fig. 1). It showed bilateral, multifocal, reversible vasoconstriction of the anterior cerebral artery, leading to a diagnosis of RCVS. After 4 months of regular monitoring, medication with pregabalin (Lyrica, 150 mg), oxycodone HCl/naloxone (Targin, 10/5 mg), alpram (0.5 mg), and milnacipran (Ixel, 25 mg) was maintained at each visit without any other procedures. The patient continues to report a feeling of heaviness around the forehead, and maintains an NRS score of 1–2.

## 3 Discussion

According to The International Classification of Headache Disorders 3rd edition (ICHD-3), Thunderclap headache is defined as sudden pain of maximum intensity occurring in less than one minute and lasting more than five minutes. Thunderclap headache should immediately carry out various neurological tests because it can be accompanied by life-threatening brain diseases. Among diseases with Thunderclap headache, RCVS is caused by causes such as Sexual activity, Valsalva manuver, lasting more than 1 month and usually decimation of symptoms within 3 months.^[5]^

RCVS is a type of secondary headache that causes sudden, severe pain. As it is not a common disease, it is difficult to know the exact incidence, but the number of diagnosed cases are gradually increasing due to advances in non-invasive CT and MRI.^[6]^ The clinical presentation of RCVS makes it difficult to differentiate from other diseases, but this patient showed consistent findings in terms of symptoms and brain CTA imaging, indicating a high likelihood of RCVS. In 30% of the cases, RCVS develops without any trigger, but it can be initiated by alcohol, sexual intercourse, triptan preparation, and the Valsalva maneuver.^[7–10]^ In the present case, the patient later developed headaches at regular intervals without a trigger. However initially, headaches occurred along with a rise in blood pressure due to the Valsalva maneuver during defecation.

RCVS is rare and difficult to differentiate with the help of symptoms alone; however, if not treated, the headaches can cause fatal complications. Among reported cases, there have been several patients experiencing complications, including anterior cerebral artery dissection 1 week after the onset of RCVS,^[11]^ convexal subarachnoid hemorrhage,^[12]^ and lobar intracerebral hemorrhage.^[12]^ This demonstrates the importance of identifying thunderclap headache when it occurs.

In terms of symptoms, RCVS headaches typically show a throbbing, pulsatile, or pounding pattern, can be accompanied by nausea or vomiting, and affect the whole of the head or both sides of the head.^[13]^ The patient in our report complained of sudden, severe throbbing and pulsatile pain on both sides of the head during defecation, and also vomited once. However, the symptoms for migraine are similar, and so it is difficult to differentiate between RCVS and migraine.^[14]^ Therefore, radiological techniques also need to be included in the diagnosis to facilitate differentiation.

Typically, RCVS shows radiological findings of diffuse segmental arterial constriction in MRA and conventional 4-vessel angiography, no specific findings in blood analysis, and elevated WBC, RBC, and protein levels in the CSF in a lumbar puncture examination.^[8]^ The patient in our report developed a headache, and showed mild stenosis in the right carotid bulb in initial brain CT and head and neck MRA scans. However, these findings could not be considered neurologically significant, and blood analysis and CSF were both normal. Nevertheless, in brain and neck CTA taken one month later, the patient showed bilateral, multifocal vasoconstriction of the anterior cerebral artery, indicating a high likelihood of RCVS.

There are several theories regarding the pathophysiology of RCVS, but vasoconstriction due to vascular tension is a core part of it, and this is known to be affected by various factors, including the diversity of the adrenergic cerebral tone response, BDNF gene polymorphism, and endothelial dysfunction caused by oxidative stress. Although there are reports that vasoconstriction does not necessarily result in headaches,^[2,8,15,16]^ the precise mechanisms or causes of headaches have not yet been revealed.

Among the patient's symptoms, the headache was most severe bilaterally, and this is also the most commonly reported symptom in RCVS.^[8]^ However, when a trigger point develops in the temporalis muscle, it causes headache or referred pain in the face, and as this can be caused by a number of different factors, we hypothesized that this patient could also have developed bilateral pain due to the formation of temporalis muscle trigger points. When we administered TPI in the temporalis muscle for the purpose of diagnostic treatment, the patient's therapeutic satisfaction was high, suggesting that RCVS may have been accompanied by myofascial pain syndrome in the temporalis muscles.^[17–19]^

Another disease that cannot be overlooked is migraine. This is due to the facts that migraine is difficult to clearly differentiate by symptoms alone, has a high incidence of 10% to 12% worldwide, affecting 28 million individuals in the US, and that there have been reports of cases in which migraine patients showed multifocal cerebral vasoconstriction in MRI.^[20–22]^ However, it is essential to differentiate migraine and RCVS, since the triptan class drugs used in acute migraine are vasoconstrictors that actually aggravate RCVS. Although the patient in our report showed similar symptoms to that of migraine, based on radiological findings and the pattern change, in which the headache was initially caused by defecation and later developed a regular pattern, RCVS could be considered to be more likely than migraine.

Based on vasoconstriction, calcium channel blockers like nimodipine and short-term glucocorticoids are reported to be effective,^[9,12,23,24]^ but since their effectiveness is uncertain, precise treatment methods are still under debate. The patient in this report did not use nimodipine but showed improvements in her headache after 3 months. In the case of RCVS, it is better not to use beta blockers because they can aggravate the illness by causing vasospasm;^[13]^ but for the patient in this case, we could not completely exclude migraine and, given that she showed psychiatric anxiety symptoms such as somatic symptom disorder, we decided to administer beta blockers. Since the patient did not show any signs of aggravation, we decided to continue the treatment.

## 4 Conclusion

RCVS is not a commonly seen disease and is difficult to diagnose; but because it can cause fatal complications, such as cerebral hemorrhage or cerebral infarction, continual monitoring is essential. Sudden thunderclap headache is likely to be a red flag and necessitates immediate neurological and radiological examination. The vasoconstrictive drugs used in general migraine aggravate RCVS even further, and so it is important to differentiate it from symptoms of migraine. In cases such as this one, where TPI in the bilateral temporalis muscles alleviates symptoms, it is likely that the headache is also accompanied by myofascial pain syndrome in the temporalis muscle, and so diagnostic treatment using this method should also be considered for other headaches.

## Author contributions

**Conceptualization:** Sie Hyeon Yoo.

**Investigation:** Jae young Ji, Ho Soon Jung.

**Methodology:** Ho Soon Jung, A Joo Kim.

**Project administration:** A Joo Kim.

**Resources:** Hee Dong Son.

**Software:** Hee Dong Son.

**Supervision:** Sie Hyeon Yoo.

**Writing – original draft:** Jae young Ji, Ho Soon Jung, Hee Dong Son.

**Writing – review & editing:** Sie Hyeon Yoo.

Ho Soon Jung orcid: 0000-0001-7220-3868.
